# Widespread SARS‐CoV‐2 Transmission Despite Limited Reported Cases and Clinical Disease: Exploring the Role of Pre‐Existing Humoral Immunity to SARS‐CoV‐2 in Eastern Sierra Leone

**DOI:** 10.1002/iid3.70463

**Published:** 2026-05-06

**Authors:** Robert J. Samuels, Nell G. Bond, Ibrahim Sumah, Donald S. Grant, Mohamed S. Kamara, Lydia Bazzano, Camilo Fernandez, Rodrigo Borrega, Sruti Chandra, Celia R. Glezer, John S. Schieffelin, Troy D. Moon

**Affiliations:** ^1^ Kenema Government Hospital Kenema Sierra Leone; ^2^ Tulane University School of Medicine New Orleans Louisiana USA; ^3^ Tulane University Celia Scott Weatherhead School of Public Health and Tropical Medicine New Orleans Louisiana USA

**Keywords:** COVID‐19, cross‐reactivity, human coronavirus, pre‐existing immunity, SARS‐CoV‐2, Sierra Leone, sub‐Saharan Africa

## Abstract

**Background:**

Since December 2019, SARS‐CoV‐2 has infected over 700 million people and caused > 7 million deaths. While much of the global north was severely affected, sub‐Saharan Africa was relatively spared. Possible reasons include a younger population, fewer comorbidities, and pre‐existing immunity. Here, we expand on previous work through a cohort of 306 subjects, with samples drawn pre‐pandemic, intra‐pandemic, and post‐vaccination.

**Methods:**

We assessed antibody reactivity to seasonal coronaviruses, emerging coronaviruses, and SARS‐CoV‐2 spike (S), nucleoprotein (N), and receptor binding domain (RBD) proteins in a longitudinal cohort. Antibody neutralization was measured using a pseudovirus neutralization assay.

**Results:**

Our data show that 16%–20% of pre‐pandemic samples had reactivity to SARS‐CoV‐2 N protein. Further, we noted relatively high reactivity to SARS‐CoV‐2 RBD (~31%), and low, but notable, seropositivity to SARS‐CoV‐2 S protein (3.3%–3.4%). We additionally found a significant jump in seropositivity to all SARS‐CoV‐2 proteins by March 2022 (intra‐pandemic), and high levels of neutralization in the intra‐pandemic samples compared to pre‐pandemic samples. A boosting effect on SARS‐CoV‐2 Spike was observed after vaccination.

**Conclusions:**

We report widespread circulation of SARS‐CoV‐2 in eastern Sierra Leone by March 2022 despite low national reporting. Furthermore, we provide evidence of pre‐existing humoral immunity to SARS‐CoV‐2 as compared to US controls. This may have resulted in less severe disease, less COVID‐19 testing and the apparent lack of clinical cases. Finally, we show boosting of SARS‐CoV‐2 Spike following vaccination. Studies comparing HLA type between symptomatic and asymptomatic cases are being planned. Studies to better characterize cellular immunity from pre‐pandemic timepoints should also be prioritized.

## 1 Introduction

As of April 2025, there have been approximately 777 million confirmed cases, and over 7 million deaths reported for COVID‐19 globally. Less than 2% of cases and deaths have occurred in Africa [1, 2]. Several health system and sociodemographic factors have been hypothesized to contribute to the lower numbers of cases and deaths, including Africa's limited testing capacity and laboratory infrastructure, host genetic factors, a younger population, potential cross‐reactivity between SARS‐CoV‐2 and other infectious diseases, fewer pre‐existing comorbid conditions like hypertension, diabetes mellitus, and obesity, as well as earlier implementation of preventive measures resulting from prior outbreak responses [2, 3, 4, 5]. Factors that may have contributed to the apparent protection from severe COVID‐19 disease in much of sub‐Saharan Africa, prior to the introduction of vaccine campaigns, are not well understood and require additional investigation.

Sierra Leone, a small West African country with an estimated 2023 population of 8.8 million persons, serves as a model for exploring these unresolved questions. As of October 2024, Sierra Leone had officially reported just 7985 cumulative cases of COVID‐19 and only 125 confirmed COVID‐19‐related deaths (case fatality rate = 1.45/100,000 population) [1, 6]. Despite a weak health system, in terms of both physical and human resources following the years of civil war and the 2014–2016 West African Ebola Outbreak, Sierra Leone's implementation of national COVID‐19 mitigation strategies was relatively fast, possibly as a result of strong pandemic preparedness plans put into place following the outbreak [7]. Anecdotal discussions with Sierra Leone's front‐line health workers underscore that sufficient COVID‐19 tests were available and that hospitals were not inundated by patients with severe respiratory symptoms, as was seen in other parts of the world. Further, Sierra Leone did not begin rolling out COVID‐19 vaccine campaigns until March 2021. Though once started, vaccine uptake was good and by December 2022, roughly 70% of Sierra Leone's adult population had been fully vaccinated [8]. Of note, mass vaccination campaigns began in Eastern Province, Sierra Leone in March 2022.

One hypothesis explaining the low burden of COVID‐19 disease in countries like Sierra Leone is the potential for pre‐existing immunity to SARS‐CoV‐2. Speculation as to the differences in the global distribution of common cold human coronavirus (HCoV) strains, or possibly, as yet unidentified HCoV strains in Africa, might correlate with some level of pre‐existing protection that could have resulted in lower detection of COVID‐19 cases or decreased severity in COVID‐19 disease [9, 10]. However, the relationship between this “pre‐existing immunity” and its impacts on SARS‐CoV‐2 remains unclear. Previous studies have looked at antibody responses to HCoV and SARS‐CoV‐2 in a variety of different ways to try and understand the interactions between them and their influence on disease severity. For example, some studies have attempted to describe whether antibodies to specific HCoVs are cross‐reactive with SARS‐CoV‐2 and provide cross‐neutralization capacity. Other studies have explored whether antibodies specific to HCoVs are boosted following SARS‐CoV‐2 infection, and how this immunity could affect the outcome of SARS‐CoV‐2 infection [11, 12, 13, 14]. Further still, some groups have explored the homology of SARS‐CoV‐2 with other beta coronaviruses and its impact on the induction of SARS‐CoV‐2 cross‐reactive antibodies and their relevance, yet results have been inconsistent and at times conflicting [11, 12, 15]. Shortcomings noted in the literature include the use of different assays to test samples, and testing samples from different, rather than the same, individuals both before and during the COVID‐19 pandemic [11, 16].

Preliminary data by our group conducted in 2020 showed that roughly 30%–80% of Sierra Leonean sera collected pre‐pandemic (2016/2017) had a positive antibody reaction to the N protein of three of the known common cold HCoVs (HCoV‐229E, HCoV‐NL63, and HCoV‐OC43) and had a statistically significant higher antibody reactivity to the N protein of SARS‐CoV‐2, SARS‐CoV, and MERS‐CoV, as compared to normal US controls [17].

We sought to validate these results by measuring the prevalence of antibody responses to the common cold HCoVs and emerging coronaviruses in a larger number of patients that were selected from an established cohort of Sierra Leoneans. Samples were collected at four timepoints: pre‐pandemic (early 2017 and late 2019), intra‐pandemic (March 2022, just prior to vaccination), and 1 month post vaccination (April/May 2022). In addition, testing was done to determine levels of serum neutralizing antibodies against SARS‐CoV‐2 both pre‐ and intra‐pandemic. Finally, immunologic responses by our Sierra Leone cohort were compared to a US‐based control group in samples collected prior to the pandemic.

## 2 Methods

### 2.1 Study Design and Population

**Figure 1:**
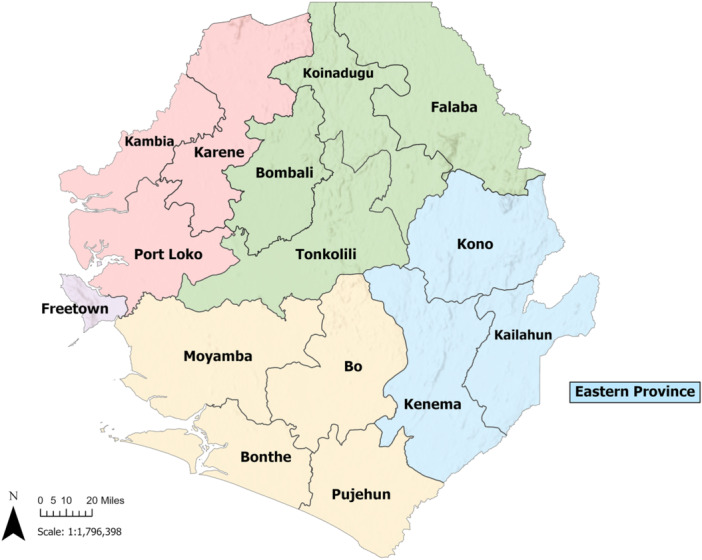
Map of Sierra Leone. Map of Sierra Leone is shown with the Eastern Province and study location highlighted.

We conducted a prospective, longitudinal cohort study in Eastern Province, Sierra Leone to understand the potential role, if any, that pre‐existing humoral immunity may have played in the comparatively lower COVID‐19 confirmed cases and associated disease severity in Sierra Leone (Figure 1). Eligible participants were selected from an established cohort of survivors of viral hemorrhagic fever (VHF), and their household contacts, residing in Eastern Province, Sierra Leone, and that had stored blood samples collected on or before December 2019, prior to the 2020 onset of the SARS‐CoV‐2 pandemic. Banked pre‐pandemic samples had been collected at two different timepoints, June–August of 2017 and March–May of 2019. This cohort consists of 2010 participants that were between the ages of 6 and 88 years of age at enrollment into this study [18]. From this cohort of participants, 306 were ultimately enrolled. The mean age of participants enrolled into the study was 25.2 years (±11.6), 58.8% were female, and all were of African origin. Here forward, we will refer to them as the VHF survivor cohort.

Blood samples that were collected between 2013 and 2016 as part of the Bogalusa Heart Study (BHS) in Bogalusa, Louisiana were used as a US‐based pre‐pandemic comparator group [19]. In total, 192 of the 1298 available samples were randomly selected for testing. Participants in the BHS were adults with a mean age of 45.3 (±4.5) years, 58.9% were female, and 34.5% identified as Black. These samples were only utilized as a laboratory comparison and not assessed for further demographic and clinical information in this study.

All individuals in the VHF survivor cohort, with stored samples collected on or before December 2019, and who were eligible to receive a COVID‐19 vaccine were eligible for inclusion in this study. In contrast, patients were excluded if they had a known prior or current infection with SARS‐CoV‐2 as determined by history and/or RT‐PCR collected on the day of enrollment; if they had previously been vaccinated against COVID‐19 or were deemed not eligible to receive a COVID‐19 vaccine.

### 2.2 Study Procedures

Eligible participants were approached for enrollment in this study, by convenience, in coordination with a community‐based national COVID‐19 vaccine campaign in March 2022. Following enrollment, paper‐based questionnaires were administered to adult participants or the parents/guardians of patients < 18 years of age. Questionnaires collected basic sociodemographic information, history of prior COVID‐19 exposure, prior COVID‐19 vaccination, and other associated clinical information. Data from these questionnaires were transferred to an electronic database utilizing the *Research Electronic Data Capture (REDCap) system* and housed at a server at Tulane University.

**Figure 2:**
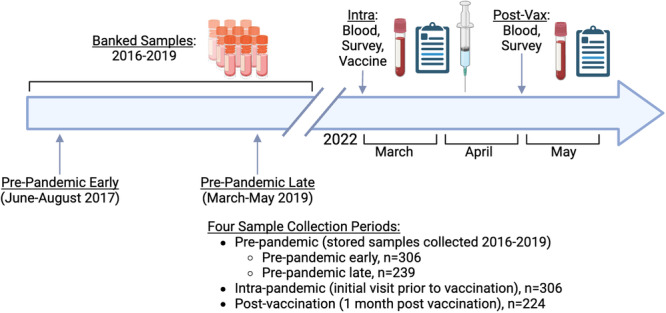
Chronology of sample collection. Schematic showing the timeline of blood sampling, questionnaire administration, and vaccination.

Next, patients had a nasopharyngeal (NP) swab collected for conduct of a COVID‐19 RT‐PCR diagnostic test as well as collection of a “intra‐pandemic” blood sample. The blood sample was collected just prior to vaccine administration with the Johnson and Johnson COVID‐19 vaccine (Janssen Biotech Inc.). Participants that were subsequently determined to be COVID‐19 negative and asymptomatic at this visit, were requested to participate in a 1‐month post‐vaccination follow‐up visit for collection of a 1‐month “post‐vaccination” blood draw (Figure 2).

NP swabs were placed in viral transport medium and blood samples were processed according to standard procedures. Both samples were stored on ice while in the field. At the end of each day, samples were processed at the Kenema Government Hospital (KGH) Biosafety Level (BSL)−3 research laboratory. NP swabs were stored in a −80°C freezer until COVID‐19 testing could be performed at the KGH laboratory. Serum aliquots were stored in a −80°C freezer at the KGH BSL‐3 laboratory until shipment, on dry ice, to Tulane University in New Orleans, Louisiana.

### 2.3 Laboratory Procedures

#### 2.3.1 Nasopharyngeal Swabs (at the KGH Lab)

NP swabs were tested for SARS‐CoV‐2 by RT‐PCR to rule out active infection. SARS‐CoV‐2 RT‐PCR testing was done according to the Sierra Leone Ministry of Health protocols. RNA extractions were performed using Qiagen RNEasy Viral RNA Extraction kits (Qiagen; Hilden, Germany). Following extraction, samples were assayed using the Sansure detection kit on a LightCycler 96 instrument (Roche, Basel, Switzerland) according to manufacturer's instructions. Data were analyzed on LightCycler 96 software (Roche Diagnostics, Basel, Switzerland). Samples were considered positive if below a Ct threshold of 36.

#### 2.3.2 Blood Samples (at the Tulane Lab)

Banked “pre‐pandemic” blood samples; prospectively collected “intra‐pandemic” and “post‐vaccination” samples; and comparator US samples were analyzed to determine (1) mean antibody concentration of the known seasonal human coronaviruses (HCoV‐OC43; HCoV‐NL63, HCoV‐299E or HCoV‐HKU1) or one of the emerging coronaviruses (MERS, SARS, and/or SARS‐CoV‐2); and (2) whether identified antibodies had neutralizing activity against SARS‐CoV‐2 at any timepoint.

#### 2.3.3 Human Coronavirus (HCoV) Antibody Testing

Antibodies were assessed using the V‐plex COVID‐19 Coronavirus (IgG) Panel 3 from Meso Scale Discovery (Meso Scale Diagnostics LLC, Rockville, MD). These multiplex IgG detection kits assessed antibody concentration of the spike protein from seasonal coronaviruses (229E, OC43, NL63, and HKU1), emerging coronaviruses (SARS‐CoV1 and MERS‐CoV), and to spike (S), nucleoprotein (N), and receptor binding domain (RBD) from SARS‐CoV‐2. Samples were diluted 1:5000 in provided sample diluent as recommended. Assays were performed according to manufacturer's instructions with attention to recommended best practices. Plates were then read immediately on a MESO QuickPlex SQ 120MM instrument (Meso Scale Diagnostics LLC, Rockville, MD) and antibody concentrations were calculated using the Meso Scale Discovery Workbench Software (version 4.0 for Windows). Clinical seropositivity cutoffs for SARS‐CoV‐2 proteins were set by the manufacturer at 1986, 5000, and 528 AU/mL for S, N, and RBD, respectively. Seropositivity cutoffs are not available for the seasonal coronaviruses, MERS, or SARS‐CoV‐1 and thus antibody concentrations are reported for these viruses.

#### 2.3.4 SARS‐CoV‐2 Pseudovirus Neutralization Assay

Serum antibody neutralization potential was measured using an established pseudovirus neutralization assay as previously described [20]. Briefly, pseudoviruses were produced by co‐transfection of 293T cells with a plasmid expressing full‐length spike protein of the Wuhan‐1 (WT) strain, containing the D614G amino acid chain (VRC7480.G614), a pCMVΔR8.2 lentivirus backbone plasmid (VRC5602), the VRC5601 plasmid pHR’ CMV Luc containing the firefly luciferase reporter gene17, and VRC9260 for TMPRSS2 expression [21, 22]. Virus stocks were collected 3 days after transfection, clarified, passed through a 0.45 μm filter, and stored in aliquots at −80°C. For neutralization, a predetermined optimal dose of pseudovirus was incubated with heat‐inactivated serum diluted 1:50 for 1 h at 37°C in 96‐well tissue culture plates. CHO/ACE2 cells, suspended by the action of TrypLE enzyme, were added to wells (10,000 cells in 100 μL medium per well), with appropriate controls. After 66–72 h of incubation, the medium was removed, and 100 μL of 1:6 dilution of Promega BriteGlo in Glo lysis buffer was added. Plates were incubated for 7 min at room temperature, after which luminescence was measured in a Biotek Synergy H1 Luminometer (Agilent, Santa Clara, CA). Luminescence was measured and neutralization percentages were calculated for each sample.

### 2.4 Statistical Analysis

Demographic and questionnaire data were analyzed using SAS (Cary, NC). Analysis of laboratory data was performed using SAS and GraphPad/PRISM (LaJolla, California). Continuous variables (antibody concentrations and % neutralization) were compared using ANOVA (when > 2 comparison groups) followed by pairwise *t*‐tests (between two groups). Bonferroni correction was utilized to control for multiple pair‐wise comparisons. Categorical variables (i.e., % seropositivity) were compared using *χ* ^2^ tests.

### 2.5 Ethical Considerations

The study protocol was approved by the Sierra Leone Ethics and Scientific Review Committee (SLESRC: December 15, 2021) and the Institutional Review Boards of Vanderbilt University Medical Center (#21282) and Tulane University (2023‐398). Written informed consent was sought from all adult participants prior to the conduct of any study‐related activities. For children, if between the ages of 12–17 years, we sought written informed assent from the child plus written informed consent from their parent/guardian. If < 12 years, we sought only informed consent from their parent/guardian.

## 3 Results

In March 2022, 306 participants from the VHF survivor cohort were enrolled into the study. Of the 306 participants enrolled, 224 (73.2%) returned for the 1‐month post‐vaccination visit and provided an additional blood sample for testing. Of the 306 participants, 58.8% were female and the median age was 24.5 years [IQR: 22–35, range: 8–70]. Questionnaires designed to capture self‐reported information about one's clinical symptoms since the start of the COVID‐19 pandemic, any hospital admissions, and any prior SARS‐CoV‐2 testing were administered. Only five participants (1.6%) reported having had any respiratory symptoms that could be consistent with COVID‐19 since the pandemic started. Of these, four participants reported that they had been tested for SARS‐CoV‐2, and only one of these reported having a positive test result. This paucity of experienced respiratory symptoms and subsequent COVID‐19 testing preclude further analysis of our data based on clinical outcomes of SARS‐CoV‐2 infection or COVID‐19 disease (data not shown).

**Figure 3:**
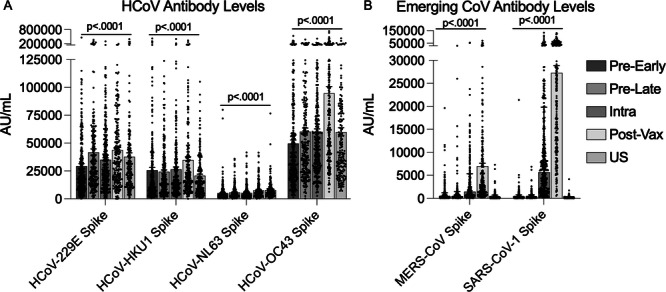
Antibody concentration of seasonal and emerging human coronaviruses. Mean antibody concentration and standard error for spike protein from seasonal HCoVs (A) and emerging CoVs (B). ANOVA testing was performed across timepoints within each virus. Pairwise comparisons can be found in Table S1.

Blood samples from each of the four collection timepoints and the US comparator samples were tested for antibodies to seasonal and emerging coronaviruses. First, upon testing for the spike protein of seasonal HCoVs, we found evidence of intermittent circulation of all four of the known HCoVs (229E, HKU1, NL63, and OC43), with significant fluctuation in the mean antibody concentration over time for each virus (*p* < 0.0001) (Figure 3A). Pairwise comparisons between timepoints showed significant differences over time, most likely consistent with seasonal circulation (Table S1). NL63 antibody concentrations were notably lower than the other seasonal coronaviruses, which has been demonstrated in previous studies [23, 24], while OC43 antibody concentration was significantly higher at the 1‐month post‐vaccination visit (96,307.79 AU/mL compared to 60,276.19 AU/mL, *p* < 0.0001). Overall, mean HCoV antibody levels were similar between the Sierra Leonean samples and the US samples across timepoints with minor exceptions: mean NL63 antibody concentration was significantly higher in the US cohort compared to SL intra‐pandemic (*p* < 0.0001); and both HKU1 and OC43 mean antibody concentrations were significantly higher in the Sierra Leonean post‐vaccination timepoint compared to the US timepoint (*p* < 0.0001 for each comparison).

**Table 1:** SARS‐CoV‐2 seropositivity.

	Pre‐early (*n* = 306)	Pre‐late (*n* = 239)	Intra (*n* = 306)	Post‐vax (*n* = 224)	US (*n* = 192)
Median sample date	July 7, 2017	March 22, 2019	March 7, 2022	April 26, 2022	October 7, 2014
SARS‐CoV‐2 antigen, *n* (%)					
Nucleocapsid	49 (16.0)	50 (20.9)	248 (81.1)	171 (76.3)	11 (5.7)
S1 RBD	96 (31.4)	74 (31.0)	285 (93.1)	222 (99.1)	16 (8.3)
Spike	10 (3.3)	8 (3.4)	282 (92.2)	221 (98.7)	2 (1.0)

*Note:* Seropositivity cutoffs: 5000 AU/mL (nucleocapsid); 528 AU/mL (S1 RBD); 1968AU/mL (spike).

Emerging coronaviruses MERS and SARS‐CoV‐1 showed a distinct pattern from the seasonal HCoVs, with mean anti‐spike antibody concentration increasing from pre‐pandemic into the intra‐pandemic time period, followed by a further significant increase 1‐month after SARS‐CoV‐2 vaccination (*p* < 0.0001, Figure 3B). Of note, Sierra Leonean samples from all timepoints had significantly higher antibody concentration than the US pre‐pandemic samples tested with one exception: MERS antibody concentration was higher in the Sierra Leonean intra‐pandemic samples compared to US pre‐pandemic samples, but did not reach significance due to the more stringent *p* value used to control for multiple comparisons (Figure 3B and Table S1).

**Figure 4:**
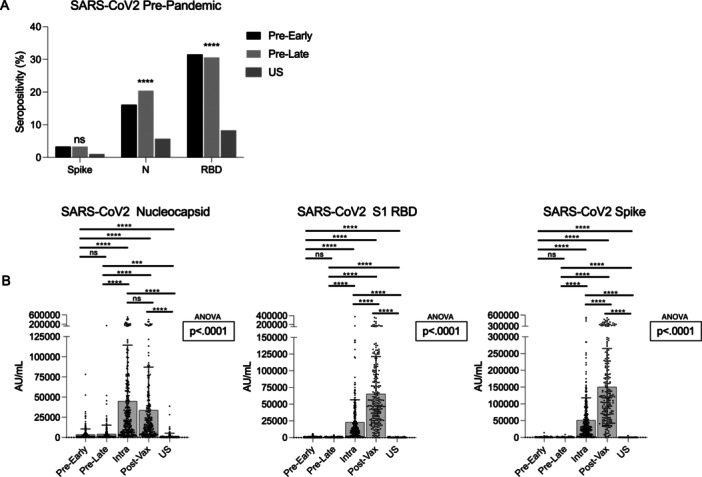
SARS‐CoV‐2 seropositivity and antibody concentration. (A) Pre‐pandemic SARS‐CoV‐2 seropositivity in Sierra Leonean Early and Late samples compared to the US samples. Chi‐square testing was done to compare seropositivity across groups. ****< 0.0001. (B) Mean SARS‐CoV‐2 antibody concentration and standard error are shown for each timepoint for N, S1 RBD, and S proteins. ANOVA was conducted to determine statistical significance across timepoints. Pairwise comparisons can be found in Table S1.

Similar to previous studies, including our own conducted in 2020, 16.0%–20.9% of study participants were seropositive for SARS‐CoV‐2 N protein prior to the COVID‐19 pandemic (Table 1 and Figure 4A). We further saw relatively high levels of SARS‐CoV‐2 S1 RBD with 31.4% and 31.0% seropositivity in the pre‐pandemic early and late timepoints. Additionally, there was low, but notable SARS‐CoV‐2 Spike seropositivity in both pre‐pandemic timepoints: 3.3% in the pre‐pandemic early and 3.4% in the pre‐pandemic late. Significantly higher antibody seropositivity to SARS‐CoV‐2 N and RBD was noted in the Sierra Leonean pre‐pandemic samples (both early and late) compared to the US samples (Figure 4A, *p* < 0.0001). Surprisingly, despite the relatively small number of national reported cases of SARS‐CoV‐2 in Sierra Leone, we saw a large jump in seropositivity to all SARS‐CoV‐2 proteins measured (81.1%, 93.1%, and 92.2% for N, RBD, and S, respectively) by March 2022. Mean antibody concentration to SARS‐CoV‐2 proteins was also significantly elevated when comparing pre‐pandemic and intra‐pandemic samples from Sierra Leone (Figure 4B and Table S1). Furthermore, we saw a significant boosting effect in antibody concentration for RBD and S following vaccination (*p* < 0.0001), but not in N, which is expected as the vaccine is a SARS‐CoV‐2 spike based vaccine [25, 26]. Also consistent with existing literature, particularly in individuals with mild or asymptomatic disease, N protein declined from 81.1% to 76.3% between the intra‐pandemic and post‐vaccination timepoints [27, 28, 29].

**Figure 5:**
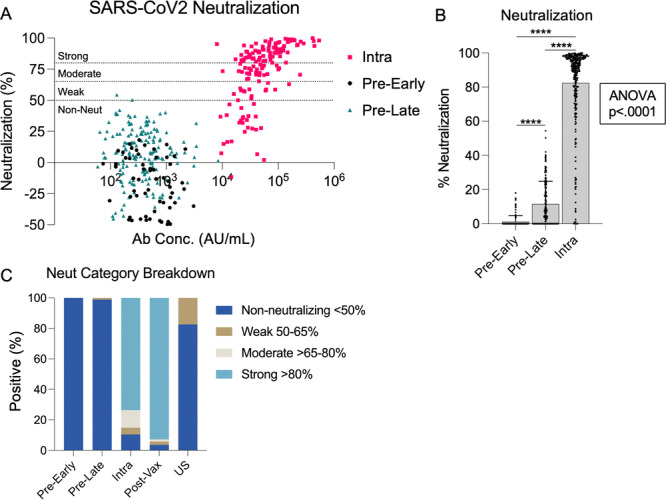
SARS‐CoV‐2 antibody neutralization. (A) SARS‐CoV‐2 % neutralization in pre‐pandemic versus intra‐pandemic samples is plotted against SARS‐CoV‐2 anti‐spike antibody concentration. The horizontal lines mark neutralization categories: non‐neutralizing (< 50%), weak (50%–65%), moderate (> 65%–80%), and strong (> 80%). (B) Mean and standard error of % neutralization for each pre‐pandemic timepoint compared to the intra‐pandemic timepoint are shown. Pairwise comparisons were conducted using unpaired *t*‐tests. ****< 0.0001. (C) Neutralization category breakdown is shown for each Sierra Leonean timepoint and the US comparison group.

Next, we measured antibody neutralization of the SARS‐CoV‐2 pseudovirus for our Sierra Leonean samples collected both pre‐pandemic and intra‐pandemic. Neutralization potential was defined as non‐neutralizing (< 50%), weak (50%–65%), moderate (65%–80%), and strong (> 80%) based on convention. We found very few samples demonstrated neutralization potential in either of the pre‐pandemic timepoints (*n* = 2, Figure 5A). Interestingly, the overall percent neutralization was significantly higher in the pre‐pandemic late timepoint compared to the early timepoint (Figure 5B). However, when categorized by strength of neutralization only two individuals from the pre‐pandemic early timepoint were categorized as weakly neutralizing, and no participants had functional neutralizing antibodies in the moderate or strong categories. Intra‐pandemic, 10.5% of participants were non‐neutralizing, 4.39% were weak, 11.5% were moderate, and 73.6% were strong neutralizers (Figure 5C). Nearly 90% of those tested intra‐pandemic were classified as moderate or strong neutralizers; comparable to previous studies which indicate a robust neutralizing antibody response to natural SARS‐CoV‐2 infection that persists for several months [30, 31]. Percent neutralization was significantly higher in the intra‐pandemic timepoint compared to each pre‐pandemic timepoint (*p* < 0.0001). Additionally, we tested the US pre‐pandemic samples for neutralization potential and found that while no participants had samples that were able to neutralize at a moderate level, 17.4% were weak neutralizers and the remaining 82.6% were non‐neutralizers.

Finally, we compared the impact of pre‐existing immunity, as defined by seropositivity to any SARS‐CoV‐2 protein, on SARS‐CoV‐2 antibody concentration and neutralization potential (Figure S1). We found that seropositivity to any SARS‐CoV‐2 protein prior to the COVID‐19 pandemic did not impact the mean antibody concentration for any SARS‐CoV‐2 protein measured either intra‐pandemic or following vaccination. Additionally, we did not observe an impact of pre‐existing immunity on percent neutralization when compared to participants who did not have evidence of pre‐existing immunity.

## 4 Discussion

Throughout the height of the COVID‐19 pandemic (2020–2022), Sierra Leone reported both a very small number of confirmed SARS‐CoV‐2 infections, as well as few COVID‐19‐related deaths [1, 6]. Early on, this finding was thought to result from Sierra Leone's weak health infrastructure and a lack of COVID‐19 testing capacity. However, as time progressed, it became clear this was not the case, as testing was recognized as readily available throughout the country. Furthermore, Sierra Leonean hospitals never experienced the same massive influx of critical respiratory illnesses, as was seen elsewhere, a finding that would not be affected by a lack of testing capacity alone [32, 33].

Our findings reveal several interesting points with regards to Sierra Leone's low number of reported cases and apparent low levels of severe disease and death. First, in Sierra Leone's Eastern Province, where our participants were recruited, samples that were collected “intra‐pandemic” showed a large jump in seropositivity to all the SARS‐CoV‐2 proteins (spike, N, and RBD) as compared to the pre‐pandemic samples. Additionally, we found that in our cohort over 90% of intra‐pandemic samples had moderate to strong neutralizing capacity against SARS‐CoV‐2. The presence of functional neutralizing antibody provides further support that these were true infections. While it is known that seasonal HCoVs, emerging CoVs, and SARS‐CoV‐2 share antigens important to the development of immune memory, the pseudovirus used to test neutralization here contains only the SARS‐CoV‐2 glycoprotein which is unique and does not contain proteins shared across CoVs. Thus, the observed responses in the intra‐pandemic and post‐vaccination timepoints are indeed confirmed SARS‐CoV‐2 responses. This highlights that by March 2022, SARS‐CoV‐2 was widely circulating and that large numbers of our participants had likely been exposed to the SARS‐CoV‐2 virus. Second, our findings reveal that 20% and 31% of our Sierra Leonean participants had cross‐reactive humoral immunity against SARS‐CoV‐2 N and RBD proteins, respectively, prior to the onset of the COVID‐19 pandemic. This pre‐existing cross‐reactivity has been described elsewhere in sub‐Saharan Africa and Asia [34, 35], and like in those studies, we presume it is due to prior infections with the four known seasonal common cold HCoVs, or potentially, an as yet unidentified coronavirus circulating in Sierra Leone. Several studies note that pre‐existing HCoV antibodies may impair SARS‐CoV‐2‐specific responses [9, 10]. Our data indicate at least that we do not see an increase in disease severity in those with pre‐existing antibodies as nearly the entire cohort did not report clinical disease. While it remains unclear whether pre‐existing cross‐reactivity to SARS‐CoV‐2 proteins provides any protective immunity, the near universal lack of reported respiratory symptoms in our cohort, despite the seropositivity to SARS‐CoV‐2 proteins, seems to support protection against disease severity at a minimum.

Interestingly, some participants who were SARS‐CoV‐2 antibody positive intra‐pandemic, did not have neutralizing antibody to our spike‐expressing pseudovirus assay. Antibodies are known to have multiple functions including neutralization and the recruitment of innate immune cells (NK cells, neutrophils, monocytes/macrophages, dendritic cells, and the complement system) [36, 37, 38]. It is likely that the antibody seropositive non‐neutralizers have functional profiles beyond neutralization rather than no antibody function whatsoever. Studies are currently under way to understand the breadth of antibody functionality in these persons including additional FC‐mediated effector functions (i.e., antibody‐dependent cellular cytotoxicity, complement deposition, etc.).

Pre‐pandemic, we showed variable levels of anti‐S antibodies to the four seasonal HCoV's (229E, HKU1, NL63, and OC43), likely consistent with seasonal circulation patterns. Interestingly, we found that mean antibody concentration for emerging CoV's (MERS‐CoV and SARS‐CoV‐1) and SARS‐CoV‐2 proteins, was significantly higher in our Sierra Leone cohort compared to the US cohort during the pre‐pandemic period, suggesting there may have been a novel CoV circulating in Sierra Leone prior to the COVID‐19 pandemic. This result is consistent with previous data looking at the N protein of emerging CoV's and SARS‐CoV‐2 [17]. We showed significantly higher mean antibody concentration to OC43, MERS‐CoV, and SARS‐CoV‐1 after SARS‐CoV‐2 vaccination. Elevated OC43 antibody concentration 1 month after SARS‐CoV‐2 vaccination could indicate a potential cross‐boosting effect of the vaccine on OC43 antibodies, cross‐reactivity of the test, or that OC43 happened to be circulating in Eastern Sierra Leone at that time. For both MERS and SARS‐CoV‐1, mean antibody concentration appears to be boosted following vaccination, which is not surprising considering the known cross‐reactivity between SARS‐CoV‐2 and emerging coronaviruses.

We know from these data that SARS‐CoV‐2 was widespread in Sierra Leone during the pandemic despite a lack of reported cases, and that pre‐existing immunity did not reduce the chance of contracting SARS‐CoV‐2. However, we were unable to fully explore the impact of pre‐existing immunity on disease severity due to a lack of a control group of individuals with symptomatic disease, a rare group in Sierra Leone but one that is possible to obtain in future studies. Additional host genetic factors likely will have an impact on the development or protection from symptomatic COVID‐19 disease in this population. Augusto and colleagues recently showed that a common human leukocyte antigen (HLA) allele, HLA‐B*15:01, was protective from symptomatic COVID‐19 disease specifically via pre‐existing T‐cell responses in a large North American population primarily of European ancestry [39]. Unfortunately, for this study, we did not have access to T‐cells collected prior to the COVID‐19 pandemic. To our knowledge, HLA type and distribution in Sierra Leone and West Africa are not well understood and may play a role in the apparent lack of disease stemming from the COVID‐19 pandemic in this region. Further studies investigating genetic factors and their relationship to asymptomatic SARS‐CoV‐2 infections in West and Sub‐Saharan Africa should be prioritized.

There are limitations which must be considered when interpreting the results of this study. The participants who were selected for this study were selected based on a convenience sample from an ongoing study which may introduce selection bias. Future population‐based studies could shed further light on the true seroprevalence of SARS‐CoV‐2 in Sierra Leone. Since our cohort was made up of Ebola and Lassa fever survivors and their household contacts, there is potential confounding due to previous VHF infection. For example, these survivors could have reduced immune responses compared to their household contacts. However, we performed a subanalysis, and the survivors and household contacts had similar antibody concentrations to SARS‐CoV‐2 proteins and similar neutralization profiles both intrapandemic and after vaccination (Figure S2). Finally, we were unable to determine the impact of pre‐existing immunity on disease outcome or severity due to a lack of control group with symptomatic disease. While this is outside of the scope of this manuscript, such a group could be recruited for future studies in Sierra Leone.

In conclusion, we report widespread circulation of SARS‐CoV‐2 in eastern Sierra Leone by March 2022 despite low national reporting of confirmed cases. Furthermore, we provide evidence of pre‐existing humoral immunity to SARS‐CoV‐2 in the population during the pre‐pandemic period as compared to a US population, possibly due to prior exposure to the known HCoV's in circulation or an as yet unidentified circulating HCoV. This pre‐existing humoral immunity may have resulted in lessening the severity of disease in our population, which then led to less seeking out of COVID‐19 testing, and the apparent lack of detection of clinical cases. We propose further studies focused on host genetic factors, particularly looking into the HLA type of individuals who presented with symptomatic disease compared to those who became SARS‐CoV‐2 seropositive without clinical disease. We intend to recruit a cohort of Sierra Leone's 7985 confirmed cases to answer this question. Finally, cellular immunity is known to be an important contributor to protective immunity in the context of SARS‐CoV‐2 [40, 41]. Although not possible with this cohort, due to a lack of banked pre‐pandemic peripheral blood mononuclear cell samples, studies investigating cellular immunity in cohorts with cryopreserved cells from pre‐pandemic timepoints should be prioritized.

## Author Contributions

**Robert J. Samuels:** conceptualization, investigation, methodology, project administration, writing – original draft, writing – review and editing. **Nell G. Bond:** formal analysis, investigation, methodology, project administration, visualization, writing – original draft, writing – review and editing. **Ibrahim Sumah:** investigation, writing – review and editing. **Donald S. Grant:** resources, writing – review and editing. **Mohamed S. Kamara:** investigation, writing – review and editing. **Lydia Bazzano:** resources, writing – review and editing. **Camilo Fernandez:** resources, writing – review and editing. **Rodrigo Borrega:** investigation, writing – review and editing. **Sruti Chandra:** investigation, writing – review and editing. **Celia R. Glezer:** investigation, writing – review and editing. **John S. Schieffelin:** conceptualization, funding acquisition, methodology, project administration, resources, supervision, writing – original draft, writing – review and editing. **Troy D. Moon:** conceptualization, funding acquisition, methodology, project administration, resources, supervision, writing – original draft, writing – review and editing.

## Ethics Statement

The study protocol was approved by the Sierra Leone Ethics and Scientific Review Committee (SLESRC: December 15, 2021) and the Institutional Review Boards of Vanderbilt University Medical Center (#21282) and Tulane University (2023‐398).

## Consent

Written informed consent was sought from all adult participants prior to the conduct of any study‐related activities. For children, if between the ages of 12–17 years, we sought written informed assent from the child plus written informed consent from their parent/guardian. If < 12 years, we sought only informed consent from their parent/guardian.

## Conflicts of Interest

The authors declare no conflicts of interest.

## Supporting information

Supporting File

## Data Availability

The data that support the findings of this study are openly available in OSF at https://osf.io/v2jm7/.
