# Supplementary material for: Widespread SARS‐CoV‐2 Transmission Despite Limited Reported Cases and Clinical Disease: Exploring the Role of Pre‐Existing Humoral Immunity to SARS‐CoV‐2 in Eastern Sierra Leone
Source: Immun Inflamm Dis. 2026 May 6;14(5):e70463. doi: 10.1002/iid3.70463 (PMC13149761; doi:10.1002/iid3.70463)
Supplement: Supplementary file 1 — Supporting File [file IID3-14-e70463-s001.docx]

**Supplemental Methods**

SARS-CoV-2 Pseudovirus Neutralization Assay: Luminescence was measured, and neutralization percentages were calculated for each sample using the following equation:

$$N=1-\left( \frac{{Lum}_{sera}}{{Lum}_{virus}} \right)$$

Where *N*=percent neutralization, *Lum_sera_*=luminescence reading of sample well including virus and serum, and *Lum_virus_*=luminescence reading of the control well with virus alone. Neutralization potential was defined as non-neutralizing (<50%), weak (50%-65%), moderate (>65%-80%), and strong (>80%) based on convention. Pseudovirus was kindly provided by Dr. James Robinson.

**Supplemental Table 1**. **Pairwise comparisons of mean HCoV antibody concentration between timepoints tested.** Mean antibody concentration in AU/mL for each comparison timepoint followed by p-value are shown. Unpaired parametric t-test with Welch’s correction. We controlled for multiple comparisons with Bonferroni correction, new significance level is: alpha/(# of comparisons)=0.05/90=0.00056. Statistically significant p-values are shown in bold. T1-T4 are from the VHF cohort from Sierra Leone. T1=Pre-Pandemic Early, T2=Pre-Pandemic Late, T3=Intrapandemic, T4=Post Vaccination. US=US Pre-Early=BHS samples.

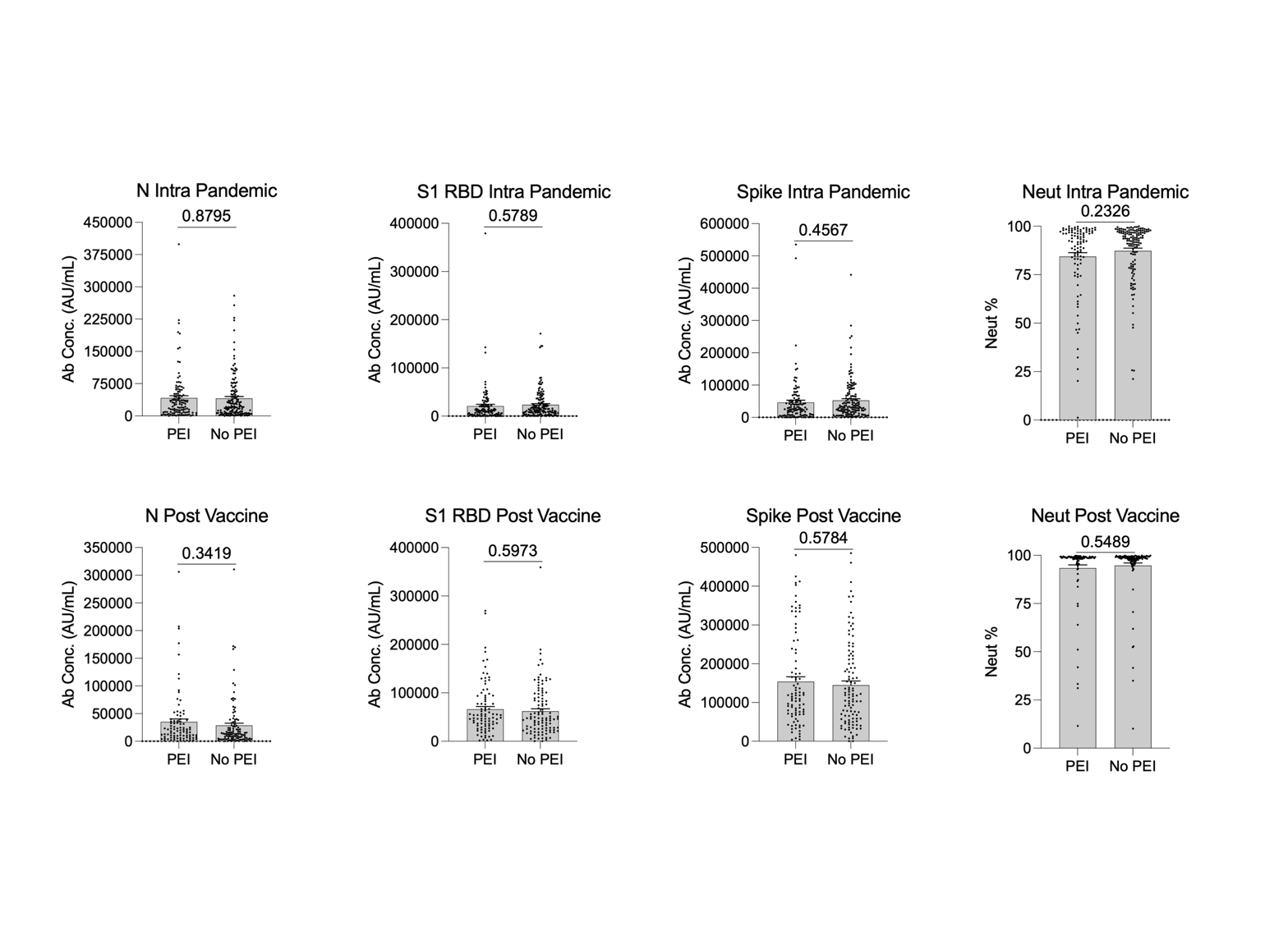


**Supplemental Figure 1. Impact of pre-existing SARS-CoV-2 seropositivity on antibody concentration and function.** Mean antibody concentration and neutralization potential are shown for participants with and without pre-existing immunity (PEI), defined as seropositivity to any SARS-CoV-2 protein prior to the COVID-19 pandemic. Error bars represent standard error. Top panel shows antibody concentration and neutralization intra-pandemic while the bottom panel represents concentration and antibody function after vaccination.


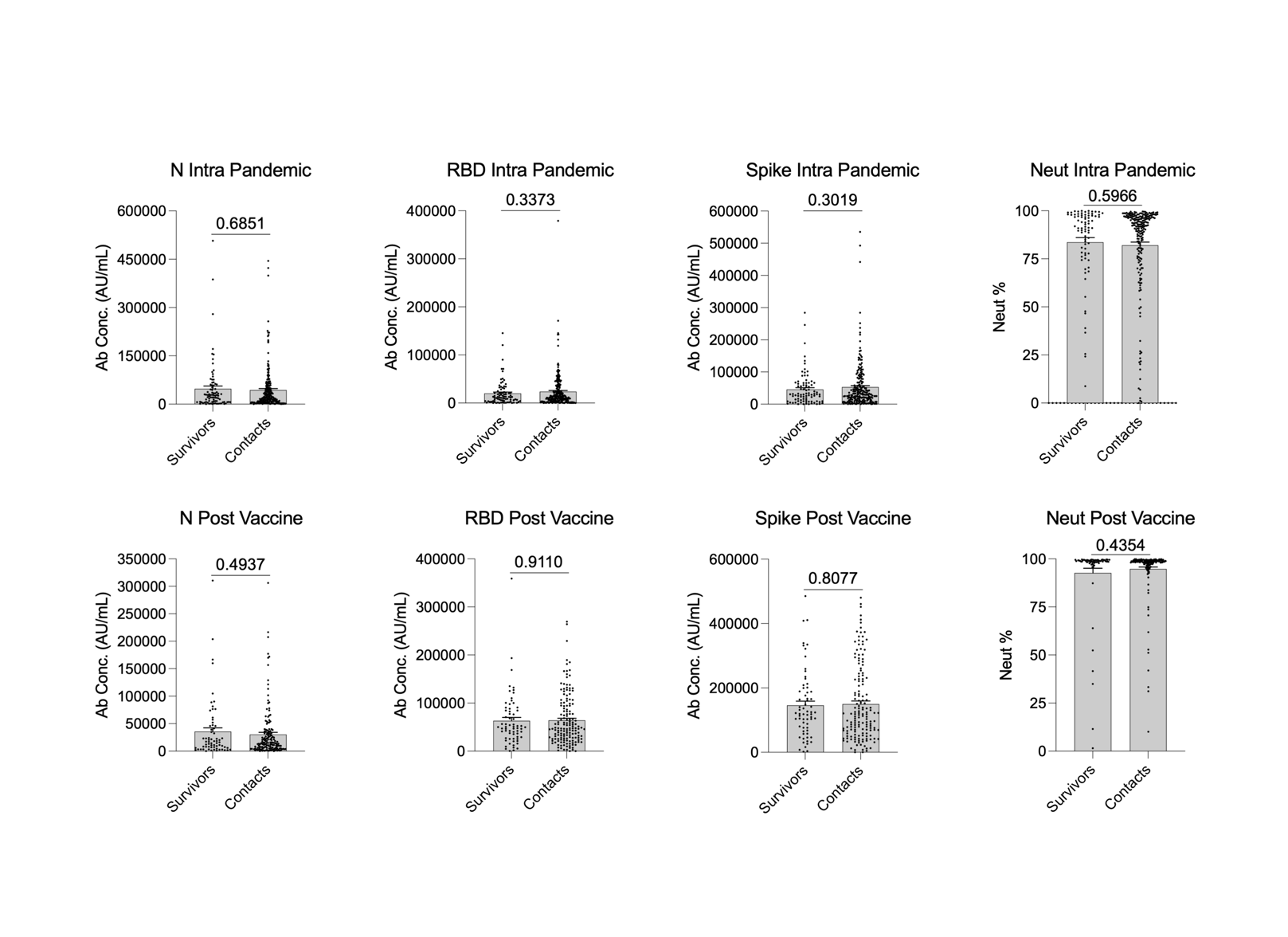


**Supplemental Figure 2. SARS-CoV-2 antibody responses are not impacted by VHF survivor status.** Mean antibody concentration and neutralization potential are shown for VHF survivor and contact participants. Error bars represent standard error. Top panel shows antibody concentration and neutralization intra-pandemic while the bottom panel represents concentration and antibody function after vaccination.
